# CD133 ^+^ progenitor cells promote pulmonary hypertension through CXCR4 signaling

**DOI:** 10.64898/2026.07.29.741640

**Published:** 2026-08-02

**Authors:** Zhiqing Wang, Dan Yi, Xinyi Zhang, Jingbo Dai, Xianming Zhang, You-Yang Zhao, Zhiyu Dai

## Abstract

**Background:**

Pulmonary hypertension (PH) is characterized by pulmonary vascular remodeling and smooth muscle cell accumulation, but the progenitor-like cells that contribute to this process remain incompletely defined.

**Methods:**

We combined analyses of human pulmonary arterial hypertension lungs and experimental PH models with bulk and single-cell RNA sequencing, lineage tracing, inducible ablation of CD133 ^+^ cells, and conditional deletion of Cxcr4 in CD133 ^+^ cells.

**Results:**

CD133 expression was markedly increased in human and experimental PH lungs. Transcriptomic analyses identified inflammatory, metabolic, chemokine-associated, and smooth muscle cell-like programs in CD133 ^+^ cells from PH lungs. Lineage tracing showed that CD133 ^+^ cells contributed to endothelial and smooth muscle cell compartments during experimental PH. Genetic ablation of CD133 ^+^ cells attenuated hypoxia-induced PH and pulmonary vascular remodeling, whereas Cxcr4 deletion in CD133 ^+^ cells reduced PH severity.

**Conclusions:**

CD133 ^+^ progenitor cells are functional contributors to pulmonary vascular remodeling, and CXCR4 signaling mediates their pathogenic activity. Targeting pathogenic CD133 ^+^ cell states or CXCL12/CXCR4 signaling may provide a strategy to limit vascular remodeling in PH.

## Full Text Availability

The license terms selected by the author(s) for this preprint version do not permit archiving in PMC. The full text is available from the preprint server.

